# Does diabetes mellitus comorbidity increase the risk of drug-induced liver injury during tuberculosis treatment?

**DOI:** 10.1371/journal.pone.0286306

**Published:** 2023-05-31

**Authors:** Ivanice D. Freire, Katherine L. Fielding, David A. J. Moore

**Affiliations:** 1 TB Centre, London School of Hygiene & Tropical Medicine, London, United Kingdom; 2 Department of Clinical Research, London School of Hygiene & Tropical Medicine, London, United Kingdom; 3 Department of Infectious Disease Epidemiology, London School of Hygiene & Tropical Medicine, London, United Kingdom; Rutgers Biomedical and Health Sciences, UNITED STATES

## Abstract

**Background:**

The growing burden of diabetes worldwide is a threat to tuberculosis (TB) control. Drug-induced liver injury (DILI) due to TB drugs is a significant concern and there is currently limited evidence on the effect of diabetes on TB DILI. This study sought to investigate the effect of diabetes as a risk factor for DILI and to further study any potential co-factors.

**Methods:**

An unmatched case-control study. *Cases* were TB patients on 2RHZE/4RH presenting with DILI from 2013–2017 in Porto Alegre, Brazil. *Controls* were contemporaneous TB patients without DILI being treated in any one of the same five Porto Alegre TB clinics. The exposure variables were diabetes (main exposure variable), age, sex, alcohol misuse, human immunodeficiency virus (HIV), hepatitis C (HCV) and B (HBV) viruses, concomitant hepatotoxic drugs, other liver diseases and TB site. The outcome variable was the occurrence of DILI.

**Results:**

Odds of DILI were increased by: older age group 51–60, 61–70 and 71–93 years (adjusted OR 2.39, 95%CI 1.30–4,38; adjusted OR 4.37, 2.28–8,35; adjusted OR 12.91, 5.81–28,66, respectively), HIV positive status (adjusted OR 3.59, 95%CI 2.25–5.73), HCV positive status (adjusted OR 3.49, 95%CI 1.96–6.21) and having concurrent pulmonary and extrapulmonary TB (adjusted OR 3.16, 95%CI 1.93–5.19). Diabetes, gender, and other hepatotoxic drugs were not associated with DILI.

**Conclusions:**

This study confirms the association between TB DILI and well-known risk factors but did not demonstrate increased odds of TB DILI in patients with diabetes.

## Introduction

Globally, the prevalence of type 2 diabetes mellitus (DM) is rapidly increasing [1]. It is an important albeit neglected risk factor for tuberculosis (TB) [2]. The combination of DM and TB has been regarded as a great challenge for global TB control particularly in low and middle income countries. In Brazil, TB remains a major public health concern, with an incidence rate of 32/100,000 population in 2021 [3], and 44/100,000 population at the time of this study in 2017 [4]. In 2021, the state of Rio Grande do Sul presented an incidence rate of 41.6/100,000 population [3]. In 2017, it was 41/100,000 population and, in the capital, Porto Alegre, the incidence rate was a little over 80/100,000 population [5]. In 2021, the incidence rate of TB in Porto Alegre was 70.7/100,000 population [5].

Ageing, changes in lifestyle, socio-economic factors and population growth have led to an increasing type 2 DM prevalence [6], with three quarters of people with DM living in low- and middle-income countries [7]. According to the Brazilian Diabetes Association, 6.9% of the Brazilian population has DM [8]. In Brazil, DM, together with malignant diseases, cardiovascular and respiratory diseases, is responsible for 80% of mortality due to chronic diseases [9].

According to the International Diabetes Federation Diabetes Atlas [10], one in eleven (33 million) adults in South and Central America are living with DM. In Brazil, 15.7 million adults (10.5%) are now living with DM (one in ten adults) and approximately 32% of people with DM in Brazil are undiagnosed [10].

Diabetes increases the risk of developing TB by three-fold [11, 12]. It also impacts negatively on TB treatment outcomes [13] due to poor glycaemic control and suboptimal levels of anti-mycobacterial drugs [13]. Tuberculosis can temporarily impair glucose tolerance, increasing glycaemic levels [11].

The incidence of drug induced liver injury (DILI) is less than 1/10,000 to 1/100,000 for most drugs in clinical practice [14, 15]. However, while its global incidence is small, its impact is substantial. Reported risk factors for DILI include older age, female sex, human immunodeficiency virus (HIV), hepatitis C virus (HCV) and hepatitis B virus (HBV), liver comorbidities, alcohol consumption, concomitance of hepatotoxic drugs, malnutrition, and genetic factors [16–22]. Chronic HBV has been found to increase the risk of DILI by TB drugs (TB DILI) by 5.5-fold [23], HCV, by five-fold [21]; and HIV, by four to five-fold [21, 24].

DILI has been a long-standing concern during TB treatment. Rifampicin, isoniazid and pyrazinamide are hepatotoxic, with a prevalence ranging from 3–28% for idiosyncratic reactions [25] (meaning that it is the characteristics of the host, such as hypersensitivity, and not the drug that are responsible for the hepatic injury) and leading to discontinuation of therapy in up to 11% of patients [26]. The reported risk of TB DILI ranges from 0.6%-33% [18]. A meta-analysis [27] has shown a mean incidence of hepatotoxicity of 1.6% for isoniazid among 38,257 adult patients (although larger reviews have reported to be 0.1%-0.3% [28]), and an incidence of 1.1% for rifampicin [27] among 1,264 adults. In a Canadian study with 430 TB patients, the incidence of hepatitis was 0.52/100 person-months of exposure to pyrazinamide, 0.18 for isoniazid, and zero for rifampicin [29]. Pyrazinamide seems to be consistently the most hepatotoxic [16, 29] and rifampicin, the least [18, 29, 30].

As far as could be ascertained, there are no studies, Brazilian or not, on the possible association between DM and DILI during TB treatment. One study has reported DM as a risk factor for severe DILI in general [15]. Non-alcoholic fatty liver disease (NAFLD), obesity, insulin resistance, polypharmacy, high alcohol intake and HCV are all DM-associated conditions acting as co-factors for liver injury [31]. This study aimed to determine whether DM was independently associated with DILI, during first-line TB treatment; and to identify co-factors predictive of an increased risk of DM-associated DILI. The objectives were twofold: to undertake a case-control study of TB patients with and without DILI to determine the effect of DM as a risk factor for DILI; and to further investigate predictors of DILI in patients with DM and any co-factors associated with this increased risk, if identified.

We hypothesized that patients with DM taking the RHZE regimen for active TB would be more likely to develop DILI than those without DM; and any increased risk for DILI in those with DM would be associated with important co-factors that further amplify any DM-associated risk. The association between type 2 DM and NAFLD, older age and use of polypharmacy, for instance, might result in an increased risk of TB DILI.

## Methods

### Design

An unmatched case-control study was conducted in which DILI during treatment for drug-susceptible TB was the outcome of interest.

### Setting

The study was conducted in the five TB clinics of Porto Alegre, Brazil, using routinely collected data recorded in medical records and in the Information System of Special Tuberculosis Treatments (SITETB) database. The TB clinics are urban referral TB clinics in the secondary level of health care in the Brazilian public health service. Only straightforward pulmonary TB in adults uncomplicated by adverse effects is treated in the primary care clinics. According to the Brazilian guidelines, patients should be referred from their general practitioners to a TB clinic if: the diagnosis is facing difficulties; there are major side effects; serious comorbidities such as immunosuppression, chronic liver disease and chronic renal disease; cases of failure to the first-line regimen; EPTB; and TB in children.

All suspected DILI cases are routinely transferred to the TB clinics to be managed by TB physicians. Primary care clinics are not allowed to decide on any change of TB treatment.

SITETB is an electronic system for notification and management of any TB cases needing a drug regimen different from the standard drug-susceptible TB regimen in fixed dose combination, be it due to a need to individualize drug doses (as in cases of children or chronic renal disease); major side effects; or drug resistance. It also reports all cases of non-tuberculous mycobacterial disease. It was implemented in 2013 and is a valuable tool for managing cases, providing data on patients’ demographics, TB diagnosis, comorbidities, previous episodes and outcomes; and pharmacological surveillance. It is not linked to any other databases.

### Participant selection

Cases were defined as TB patients of any age who had a DILI diagnosis whilst regularly taking (no less than 15 days in any 30-day period) the standard fixed-dose drug-susceptible TB regimen (rifampicin, isoniazid, pyrazinamide and ethambutol [2RHZE/4RH]) and followed-up in any one of the five TB clinics after the DILI diagnosis. DILI was diagnosed by the attending TB physician according to the standard international consensus definition of TB DILI [18] and with a resulting change in treatment. In the absence of symptoms, the DILI definition was based on elevation of transaminases ≥5 times the upper limit of normal (ULN) and in the presence of symptoms, ≥3 times the ULN or twice the bilirubin ULN).

Control subjects were TB patients of any age treated with 2RHZE/4RH in any one of the five TB clinics, who did not develop DILI. Patients were ineligible as controls if their treatment was taken irregularly or if lost to follow up before the 120^th^ day. Candidates were selected by a systematic sample of every third patient directly from medical records using a 1:4 case:control ratio and frequency matched by calendar year. Cases and controls were identified among TB registrations from 1 January 2013 to 31 July 2017.

Patients were selected as cases if they presented the following criteria: presentation of DILI diagnosis from 01 January 2013 to 31 July 2017; patient originated from a general practitioner’s clinic, TB clinic or discharged from a hospital; registration on the Information System for Special Tuberculosis Treatments electronic platform due to DILI, from 01 January 2013 until 31 August 2017 –in so far as the DILI episode happened no later than 31July 2017—due to a need of change of treatment prescribed; follow-up in one of the five TB clinics in Porto Alegre either before the DILI episode or after that.

Patients were excluded based on the following criteria: patients missing any one of the three hepatotoxic drugs, namely rifampicin, isoniazid or pyrazinamide; patients were not excluded if they were not treated with ethambutol, as this drug is not associated with hepatotoxicity. Similarly, patients who died were not excluded.

The control subjects were TB patients who were treated with RHZE, and who did not develop DILI during the same time frame, from 01 January 2013 until 31 July 2017. The inclusion criteria were as follows: controls were chosen if they had been treated regularly for TB in any of the same five referral TB clinics during the same period. In the unlikely event of a control being registered on SITETB for any other reasons than DILI, they were still considered eligible in so far as they remained on the RHZE regimen throughout treatment, even if in the following circumstances: if ethambutol was not prescribed (pediatric patients under ten years of age) or if it was discontinued for any reason; if tablets were prescribed individually due to low weight, chronic renal failure or chronic liver disease.

Patients with the following indications for registration on SITETB were ineligible as a control: mono-resistance to isoniazid or to rifampicin, or any non-DILI side effects leading to a disruption in the RHZ combination, such as untreatable gastro-intestinal intolerance to any of them; patients who had their treatment discontinued before completing 120 days, who were lost to follow-up for whatever reason or died before the 120th day of treatment; and patients with treatment irregularity, defined as taking treatment on less than 15 days in any 30 days period.

### Data collection

Data were abstracted from the SITETB database and medical records. The main exposure of interest was DM; others were age, sex, HIV, HCV, HBV, hazardous drinking, concomitant hepatotoxic drugs, liver comorbidities and TB site. The time from TB treatment start to DILI diagnosis was also abstracted.

The diagnosis of diabetes in this clinical scenario was dependent upon identification of HbA1c ≥ 6.5% or a fasting plasma glucose ≥ 126 mg/dl, or a random plasma glucose >200 mg/dl with classical symptoms of polyuria and polydipsia, as defined by the criteria of the Expert Committee on the Diagnosis and Classification of Diabetes Mellitus American Diabetes Association [32, 33].

Both diabetes that was previously known and newly diagnosed at any point prior to or during the TB episode was included. Pre-diabetes was not included.

Patients not tested for diabetes were classified as ‘not known to have diabetes’ to reflect the inability to assign ‘no diabetes’ status to an individual who has not been tested and had a diagnosis of diabetes ruled out.

Subjects were classified as *known* or *not* known DM (acknowledging that this may have misclassified individuals with undiagnosed DM). Age groups were <39; 40–50, 51–60, 61–70 and over 70 years. Hazardous drinking was self-reported and defined as a pattern of alcohol consumption leading to adverse health events and not necessarily a dependence diagnosis and classified as *known to be a hazardous drinker* or not (including an unknown status). Patients were classified as *known to be HIV-*, *HCV-* or *HBV-*positive or not (including unknown results). Concomitant drugs were classified as hepatotoxic according to a standardized system for categorizing DILI-causing drugs based on the number of published reports of convincingly documented DILI. Drugs in categories A (≥50 reports) and B (12–49 reports) [34] were recorded as hepatotoxic. Patients were classified as *known to have another liver disease* (apart from HCV or HBV), if the diagnosis was confirmed or strongly suggested or *not known* (unconfirmed or unknown). Tuberculosis sites were: only pulmonary TB (PTB), only extrapulmonary TB (EPTB) or both PTB-EPTB.

### Data analysis

A pilot assessment of DILI cases in diabetic patients from the five TB clinics was conducted by the researchers. It was found that amongst patients on TB treatment, the prevalence of DM was 6.8% and amongst the cases (TB patients with DILI), it was 12.5%.

From these preliminary data indicating 12.5% prevalence of DM amongst DILI cases, to detect an odds ratio (OR) of 1.98 for the effect of DM on TB DILI, assuming 1:4 cases to controls ratio, a type I error of 5% and power of 80%, a target sample size of 156 cases and 624 controls was calculated.

Unadjusted odds ratios (ORs) for being a case and 95% confidence intervals (CI) were calculated by logistic regression. Confounding and interaction were initially explored using the Mantel-Haenszel analysis. Variables that resulted in a 10% or more change in the OR for DM from the Mantel-Haenszel analysis and not considered to be on the causal pathway were adjusted for in a logistic regression model. Age and sex were adjusted for *a priori*. Interaction between variables and DM, adjusting for all variables in the model, was assessed using logistic regression based on effect modification observed in the Mantel-Haenszel analysis. Time to DILI was summarized as median and interquartile range (IQR) and displayed graphically using Kaplan-Meier curves stratified by DM. The Log-rank test was used for comparing time to DILI by DM status. Statistical analysis was undertaken in Stata® V.14 (StataCorp).

The study protocol was approved by the London School of Hygiene & Tropical Medicine Research Ethics Committee and by both the Porto Alegre and the State of Rio Grande do Sul Health Department Research Ethics Committees. Requirement for patient consent was waived as the study was conducted using routine data managed and analyzed without personal identification.

### Inclusivity in global research

Additional information regarding the ethical, cultural, and scientific considerations specific to inclusivity in global research is included in the Supporting Information (S1 Checklist).

## Results

A total of 791 subjects were included in the study, 157 DILI cases and 634 controls (Table 1). Among cases, the median age was 46 years (IQR 37–60, range 1–91 years). Potentially hepatotoxic drugs were taken concomitantly by more than half of the cases (94/157, 59.9%); the most common drugs being efavirenz and trimethoprim-sulfamethoxazole, each reported for 29.8% (28/94) patients. Among cases, 17 (10.8%) had known DM, of whom 40.1% were over 50 years of age, 61.1% were male and 59.9% were taking concomitant hepatotoxic drugs.

**Table 1:** Participant characteristics of 157 TB patients experiencing DILI (cases) and 634 TB patients without DILI (controls).

Patient characteristics		Cases (DILI) n = 157 N (col %)	Controls (not DILI) n = 634 N (col %)
**Known DM**	No	140 (89.2%)	571 (90.1%)
	Yes	17 (10.8%)	63 (9.9%)
**Age group (years)**	1–39^1^	51 (32.5%)	354 (55.8%)
	40–50	43 (27.4%)	127 (20.0%)
	51–60	24 (15.3%)	74 (11.7%)
	61–70	22 (14.0%)	61 (9.6%)
	71–93	17 (10.8%)	18 (2.8%)
**Sex**	Male	96 (61.1%)	351 (55.4%)
	Female	61 (38.9%)	283 (44.6%)
**Known hazardous drinking**	No	105 (66.9%)	526 (83.0%)
	Yes	52 (33.1)	108 (17.0%)
**Known HIV infection**	No	79 (50.3%)	496 (78.2%)
	Yes	78 (49.7%)	138 (21.8%)
**Known HCV infection**	No	121 (77.1%)	598 (94.3%)
	Yes	36 (22.9%)	36 (5.7%)
**Known HBV infection**	No	152 (96.8%)	632 (99.7%)
	Yes	5 (3.2%)	2 (0.3%)
**Known hepatotoxic drugs**	No	63 (40.1%)	326 (51.4%)
	Yes	94 (59.9%)	308 (48.6%)
**Known other liver diseases**	No	135 (86.0%)	618 (97.5%)
	Yes	22 (14.0%)	16 (2.5%)
**TB site**	Only PTB	85 (54.1%)	420 (66.3%)
	Only EPTB	23 (14.7%)	145 (22.9%)
	PTB and EPTB	49 (31.2%)	69 (10.9%)

^1^ The youngest patient was 3 months old; median age (IQR) among cases and controls was 46 years (IQR 37–60) and 37 years (IQR 25–50), respectively

CI: confidence interval; col: column; DILI: drug-induced liver injury; DM: diabetes mellitus; EPTB: extrapulmonary tuberculosis; HBV: hepatitis B virus; HCV: hepatitis C virus; HIV: human immunodeficiency virus; IQR: interquartile range; OR: odds ratio; PTB: pulmonary tuberculosis

Of the 749 medical records reviewed, 634 patients fulfilled the screening criteria for being a control and were included. A total of 115 patients were excluded from being a control and the reasons were: loss to follow-up from treatment (defined as missing >30 days of treatment) before completing four months of treatment (54; 47.0%), transfer from the clinic before four months (15; 13.0%), taking treatment on ≤15 days in any 30 days period (13; 11.3%), death before four months (10; 8.7%), and others (23; 20.0%). No controls with known DM were excluded.

Among controls the age ranged from three months to 93 years old, with a median of 37 years (IQR 25–50). Nearly half of the controls (48.6%) were taking hepatotoxic drugs. Of the 634 controls, 63 (9.9%) had known DM, of whom 56% were over 50 years of age, 57% were male and 78% were taking concomitant hepatotoxic drugs. Supplementary tables present a complete drug list (S1 Table), liver comorbidities (S2 Table), and EPTB sites (S3 Table).

Diabetes prevalence was similar in cases and controls, giving an unadjusted OR 1.10 (95%CI 0.62–1.94) (Table 2). Increasing age was associated with increasing risk of DILI; a 1.5-fold increase in odds of DILI from one age group to the next (*P*<0.001). There was no evidence of an effect of sex on DILI. Hazardous drinking (unadjusted OR 2.41; 95%CI 1.62–3.59), known HIV-positive (unadjusted OR 3.55; 95%CI 2.54–5.18) and known HCV infection (unadjusted OR 4.94; 95%CI 2.95–8.29) were associated with increased odds of DILI. Also, patients taking other hepatotoxic drugs (unadjusted OR 1.58; 95%CI 1.11–2.26); patients with a liver comorbidity (unadjusted OR 6.29; 95%CI 3.17–12.51); and having both PTB-EPTB when compared to those with only PTB (unadjusted OR 3.51; 95%CI 2.27–5.42) were associated with increased odds of DILI.

**Table 2:** Univariable analysis of potential risk factors for DILI (n = 791) and multivariate logistic regression. Fully adjusted analysis of the model without interactions between the exposure variables and DILI (n = 791).

Risk Factors for DILI		UnadjustedOR	95% CI	*P—*value	Adjusted OR	95% CI	*P—*value
**Known DM**	No	1	-	0.7	1	-	0.7
	Yes	1.10	0.62–1.94	-	0.88	0.45–1.71	-
Age group (years) ^2^	1–39 ^1^	1	-	<0.001	1	-	<0.001
	40–50	2.35	1.49–3.70	-	1.69	1.00–2.86	-
	51–60	2.25	1.30–3.89	-	2.39	1.30–4.38	-
	61–70	2.50	1.42–4.42	-	4.37	2.28–8.35	-
	71–93	6.56	3.18–13.53	-	12.91	5.81–28.66	-
**Sex**	Male	1	-	0.2	1	-	0.7
	Female	0.79	0.55–1.13	-	1.09	0.72–1.63	-
**Known hazardous drinking**	No	1	-	<0.001	-	-	-
	Yes	2.41	1.62–3.59	-	-	-	-
**Known HIV infection**	No	1	-	<0.001	1	-	<0.001
	Yes	3.55	2.43–5.18	-	3.59	2.25–5.73	-
**Known HCV infection**	No	1	-	<0.001	1	-	<0.001
	Yes	4.94	2.95–8.29	-	3.49	1.96–6.21	-
**Known HBV infection**	No	1	-	0.003	-	-	-
	Yes	10.39	1.97–54.83	-	-	-	-
**Known hepatotoxic drugs**	No	1	-	0.01	1	-	0.4
	Yes	1.58	1.11–2.26	-	0.84	0.54–1.29	-
**Known other liver diseases**	No	1	-	<0.001	-	-	-
	Yes	6.29	3.17–12.51	-	-	-	-
**TB site**	Only PTB	1	-	<0.001	1	-	<0.001
	Only EPTB	0.78	0.48–1.29	-	0.75	0.43–1.30	-
	PTB and EPTB	3.51	2.27–5.42	-	3.16	1.93–5.19	-

^1^ The youngest patient was 3 months old; median age (IQR) among cases and controls was 46 years (IQR 37–60) and 37 years (IQR 25–50), respectively

^2^ Unadjusted OR 1.45 (1.27–1.66) *P*<0.001

*P*-value for the Likelihood Ratio test

CI: confidence interval; DILI: drug-induced liver injury; DM: diabetes mellitus; EPTB: extrapulmonary tuberculosis; HBV: hepatitis B virus; HCV: hepatitis C virus; HIV: human immunodeficiency virus; IQR: Interquartile range; OR: odds ratio; PTB: pulmonary tuberculosis; TB: tuberculosis

A Mantel-Haenszel analysis was performed to assess for confounding and interaction. Individually, sex, hazardous drinking and other liver diseases did not appear to confound any effect of DM on the odds of having DILI. However, age (DM M-H OR 0.72; 95% CI 0.40–1.31), HIV (DM M-H OR 1.44; 95% CI 0.80–2.60) and TB site (DM M-H OR 1.29; 95% CI 0.72–2.32) presented as confounders on the effect of DM on DILI. Bivariate analysis to explore the effect of HBV on the DM OR for DILI was not conducted due to sparse data (seven patients in total).

After controlling for HCV infection, there was weak evidence for HCV modifying the effect of DM on DILI (DM M-H OR 0.93; 95% CI 0.51–1.69; *P* = 0.06). Likewise, there was evidence that concomitant use of hepatotoxic drugs modified the effect of DM on DILI (DM M-H OR 0.98; 95% CI 0.55–1.75; *P* = 0.01). These interactions were considered in more detail in the multivariable regression model.

A multivariate logistic regression analysis was performed to obtain a fully adjusted OR for the effect of DM on DILI (Table 2). The fully adjusted model controlled for age, HIV and TB site as all were identified as important confounders in the Mantel-Haenszel analysis. The sex variable was included in the model *a priori*. In addition, HCV and hepatotoxic drugs were included in the model for completeness as they were assessed for whether they were effect modifiers below.

The adjusted OR for DM was 0.88 (95%CI 0.45–1.71), after controlling for age, sex, HIV, HCV, hepatotoxic drugs and TB site (Table 2). Even though the modelling strategy was not based on assessing the causal effect of other variables on DILI, it was noted that the following variables were strongly associated with increased odds of DILI: age over 50 years of age (*P*<0.001), being HIV-positive (adjusted OR 3.59, 95%CI 2.25–5.73), being HCV-positive (adjusted OR 3.49, 95%CI 1.96–6.21) and having both PTB and EPTB (*vs*. only PTB, adjusted OR 3.16, 95%CI 1.93–5.19).

There was some evidence that HCV modified the effect of DM on DILI (*P-*value for interaction 0.02), after adjusting for age, sex, HIV, hepatotoxic drugs and TB site (Table 3). Among those with known HCV-infection, DM was associated with a three-fold increase in the odds of DILI, whereas among those with no known HCV-infection the adjusted OR for DM was 0.5 (Table 3).

**Table 3:** Interaction between DM and hepatotoxic drugs and DM and HCV, adjusted for age group, sex, HIV, HCV, hepatotoxic drugs and TB site (n = 791).

Stratified by	DM	Cases (col %)	Controls (col %)	Stratum-specific adjusted OR for DM (95%CI)	*P*-value for interaction
**Known concomitant hepatotoxic drugs** ^1^					
**No**	No	56 (88.9%)	312 (95.7%)	1	0.1
	Yes	7 (11.1%)	14 (4.3%)	1.78 (0.62–5.12)	
**Yes**	No	84 (89.4%)	259 (84.1%)	1	-
	Yes	10 (10.6%)	49 (15.9%)	0.61 (0.26–1.40)	
**Known HCV** ^2^					
**No**	No	113 (93.4%)	539 (90.1%)	1	0.02
	Yes	8 (6.6%)	59 (9.9%)	0.54 (0.23–1.26)	
**Yes**	No	27 (75%)	32 (88.9%)	1	-
	Yes	9 (25%)	4 (11.1%)	3.37 (0.86–13.16)	

^1^ Adjusted for age group, sex, HIV, HCV and TB site

^2^ Adjusted for age group, sex, HIV, hepatotoxic drugs and TB site

CI: confidence interval; col: column; DM: diabetes mellitus; HCV: hepatitis C virus; HIV: human immunodeficiency virus; OR: odds ratio; TB: tuberculosis

After adjusting for age, sex, HIV, HCV and TB site, there was no strong evidence to assume an interaction between hepatotoxic drugs and DM (*P*-value for the interaction = 0.1). The stratum specific ORs for DM were 1.78 (95% CI 0.62–5.12) among those taking no hepatotoxic drugs, and 0.61 (95% CI 0.26–1.40) among those taking hepatotoxic drugs. In the *no hepatotoxic drug* stratum, there were only seven cases with DM.

**Fig 1:**
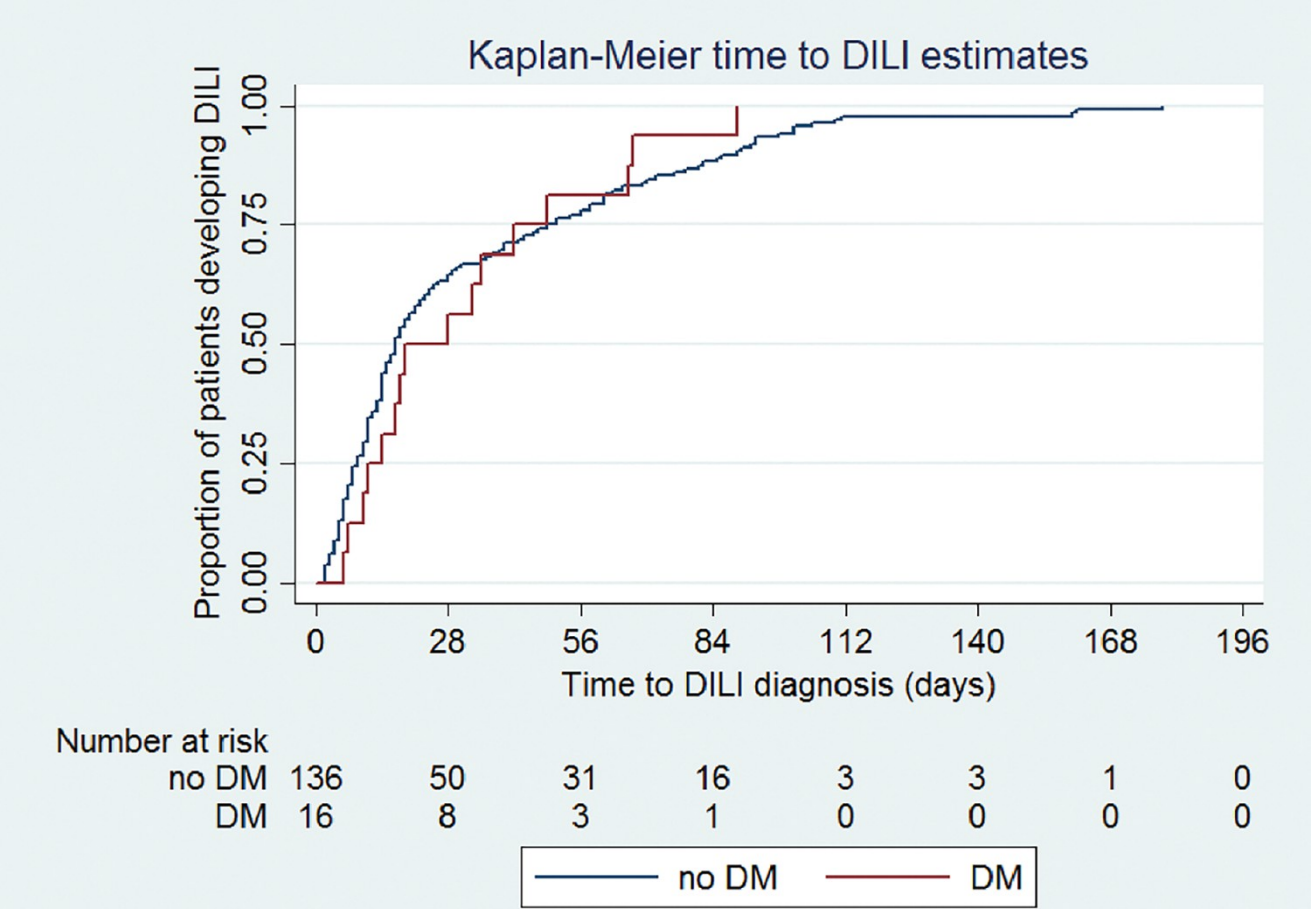
Kaplan-Meier curves for time to DILI from treatment initiation, stratified by DM status (n = 152). DILI: drug induced liver injury; DM: diabetes mellitus.

As the main exposure of interest, DM was also analysed in terms of time to DILI (Fig 1). Of the 157 cases, the time to DILI diagnosis from TB treatment initiation could be determined in 152 cases (97%); five cases could not have their exact DILI dates identified. The median time was 17.5 days (IQR 9.5–49) overall; 63.8% had DILI by 28 days and 78.3%, by 56 days. There was no association between time to DILI and DM (*P* = 0.9).

To conclude, from the present fully adjusted analysis for presenting an OR for DM on DILI, it was shown that DM did not increase the odds of having TB DILI.

## Discussion

Our study, using routine data, found no evidence that DM was associated with TB DILI. Older age, HIV, HCV and having both PTB and EPTB were shown to independently increase the odds of DILI.

To the best of our knowledge, no study has hypothesized an association between DM and TB DILI and sought to study DM as a main exposure based on a biologically plausible hypothesis. The association between type 2 DM and NAFLD, older age and use of polypharmacy, led us to consider that patients with DM could be at a higher risk of having TB DILI. The lack of a demonstrated effect from previous studies of TB-DILI risk factors could have been a reflection of limited data [16, 26, 35, 36].

The risk of TB DILI increases with age and the highest incidence occurs over 50 years of age [37]. Our study confirmed that for older patients, the odds of DILI increased and were the greatest in the older age group, in agreement with several studies [17, 26, 29, 37]. Bright-Thomas *et al*. [38] identified a similar trend, with risk increasing 16% for every 10-year increase in age (95%CI 1.02–1.32). Age over 60 years old has been independently associated with DILI (OR 3.1; 95%CI 1.6–7.6) [35] and (OR 3.5; 95%CI 1.3–10.1) [26]. Lack of adjustment for chronic conditions may partly explain study results where this association has not been found [19, 39].

Female sex was not associated with DILI, in contrast with some other studies [16, 17, 24, 35]. One study [17] has found that females were associated with a four times higher risk for severe DILI when compared to males. Others have found no such increased risk [26, 38, 40].

Alcohol consumption increases the risk of TB DILI [35, 37] with reported OR ranging from 2.2 (95%CI 1.9–5.3%) [35] to 4.76 (95%CI 2.25–10.05) [37]. However, such findings are not universal [17, 21, 26, 29, 40], possibly depending on different drinking patterns and ways of recording drinking. Not being confounders, hazardous drinking and other liver diseases (mostly cirrhosis and hepatic TB) were not included in the multivariate analysis.

The HIV-TB DILI association we found is concordant with the existing published literature [20, 21, 24, 29]. A study conducted in the United Kingdom demonstrated a four-fold increased odds of TB DILI (95%CI 1.06–18.3) for those HIV-positive *vs*. -negative [39]. The use of protease inhibitors might partially explain this tendency. Other studies, however, have found no such association [29, 35, 41].

Co-existing viral hepatitis are important risk factors for DILI [18, 21]. Many studies have reported the HCV-DILI association [21, 41, 42], although others have not [16, 39]. In our study, both the effect of HCV on TB DILI and its association with DM may have been underestimated, given that HCV status was not entirely known.

No association was found between concomitant hepatotoxic drugs and TB DILI, in agreement with other authors [35, 43]. An association was found between having both PTB and EPTB and DILI. Mostly, this reflects disseminated TB, which is frequently associated with undernutrition and HIV, both risk factors for DILI. However, this effect was still seen after adjustment for HIV. The extent of PTB has been also reported as a predictor for DILI [17, 19, 37]. A study with HIV co-infected patients has found no association between DILI and TB morbidity [43]. Conversely, a study has reported a 32% higher risk of DILI in EPTB than in PTB [24], as has been found in miliary TB (OR 2.3; *P =* 0.03) [44].

The time to DILI reported in this study agrees with the literature [36, 37, 41, 43]. It could be determined in 152 cases (97%) for which the median time was 17.5 days from initiation of treatment (IQR 9.5–49) overall; 63.8% had DILI by 28 days and 78.3%, by 56 days. Time to DILI was unaffected by DM status (*P* = 0.9). In a previous study, 72% of DILI cases occurred within the first 28 days and 87.6%, by 56 days [39], similar to our findings.

In the multivariate analysis, there was some evidence that having HCV modified the effect of DM on DILI (*P*-value for the interaction = 0.02), although this finding should be treated with caution, as numbers are small, and it was not a pre-defined hypothesis. The biological plausibility for this may lie with the potential for HCV to exacerbate DM-associated conditions, such as NAFLD, which could augment susceptibility to DILI.

To our knowledge, this was the first study to hypothesize that DM might be a risk factor for TB DILI and to seek to investigate its associations with other risk factors. Additional strengths include the SITETB database, a high HIV screening coverage, a heterogeneous study population with a wide spectrum of predictors for DILI, a variety of TB sites and disease extent and, lastly, TB physicians diagnosing and managing side effects in a standardized way.

A certain degree of misclassification bias was unavoidable. Patients who were reported as not having DM in the TB notification database and were not screened for DM later during TB treatment, were considered as *not known to have DM*. There was no demonstrable association between DM and DILI, but one cannot exclude the possibility that some undiagnosed DM subjects were included in the *not known to have* category and but for the lack of a blood test, may have been differently classified. However, we believe that represented a small underestimation of the exposure, as the prevalence of known DM in TB patients at the time, in Porto Alegre, was 6.8%.

Data reported on alcohol consumption and concomitant medications are likely to be underestimated and under-reported by patients. There were no standardized measures for alcohol units due to the retrospective nature of the data. There may have been, also, better ascertainment of some exposures, for instance, chronic viral hepatitis in DILI cases compared to those without DILI. Lastly, it was unfeasible to control for exposures which were not routinely recorded in TB practice, such as NAFLD and genetic factors.

In conclusion, this study sought to determine whether DM is associated with an increased risk of TB DILI and has found no such association. This finding does not support the diversion of scant resources available in TB control towards enhanced DILI vigilance for TB patients with DM. Increasing age, HIV, HCV, and having both PTB-EPTB independently increased the odds of TB DILI, corroborating the literature.

## Supporting information

S1 Checklist (PDF)

S1 Table Hepatotoxic drugs taken concomitantly by 402 (50.8%) patients. (PDF)

S2 Table Other liver diseases in cases and controls. ^1^ Cases: Alcoholic liver disease but not cirrhotic (1), steatosis (1), lithiasis (1), granulomatous hepatitis (1), Systemic Lupus Erythematosus (1) and choledocholithiasis (1). One patient had cirrhosis and congestive heart failure concomitantly. ^1^ Controls: Alcoholic liver disease but not cirrhotic (2), steatosis (1), autoimmune hepatitis (1), liver metastasis (1), non-alcoholic steato-hepatitis (1).
(PDF)

S3 Table Extrapulmonary TB sites (not mutually exclusive) in cases and controls. CNS: central nervous system. ^1^ Cases: peritoneum (4), bone marrow (3), testicle (2), pericardium (1), bone (1), kidney (1) and psoas (1). ^1^ Controls: eye (7), pericardium (6), peritoneum (5), testicle (3), bone (2), skin (2), joint (1), kidney (1), bladder (1), ureter (1), oesophagus (1), larynx (1), pharynx (1), vertebrae (1), intervertebral disc (1), thoracic wall (1) and bone marrow (1).
(PDF)
