# Supplementary material for: Does diabetes mellitus comorbidity increase the risk of drug-induced liver injury during tuberculosis treatment?
Source: PLoS One. 2023 May 31;18(5):e0286306. doi: 10.1371/journal.pone.0286306 (PMC10231779; doi:10.1371/journal.pone.0286306)
Supplement: S1 Table — (PDF) [file pone.0286306.s002.pdf]

Supporting information

S1 Table. Hepatotoxic drugs taken concomitantly by 402 (50.8%) patients

Drug name	Frequency	%
**Omeprazole**	118	29.4
**Efavirenz**	88	21.9
**Sulfamethoxazole with Trimethoprim**	80	19.9
**Amoxicillin with or without clavulanate**	49	12.2
**Metformin**	33	8.2
**Azithromycin**	28	7.0
**Zidovudine**	28	7.0
**Enalapril**	24	6.0
**Ibuprofen**	24	6.0
**Valproic acid**	24	6.0
**Fluconazole**	22	5.5
**Simvastatin**	18	4.5
**Captopril**	18	4.5
**Amitriptyline**	17	4.2
**Chlorpromazine**	13	3.2
**Phenobarbital**	12	3.0
**Carbamazepine**	10	2.5
**Contraceptives**	10	2.5
**Clindamycin**	9	2.2
**Haloperidol**	9	2.2
**Glibenclamide**	8	2.0
**Allopurinol**	7	1.7
**Ciprofloxacin**	7	1.7

**Imipramine**	7	1.7
**Phenytoin**	7	1.7
**Methotrexate**	5	1.2
**Sertraline**	5	1.2
**Azathioprine**	4	1.0
**Ranitidine**	4	1.0
**Sulfadiazine**	4	1.0
**Ceftriaxone**	3	0.7
**Clopidogrel**	3	0.7
**Levofloxacin**	3	0.7
**Nitrofurantoin**	3	0.7
**Paroxetine**	3	0.7
**Atorvastatin**	2	0.5
**Diclofenac**	2	0.5
**Hydralazine**	2	0.5
**Infliximab**	2	0.5
**Itraconazole**	2	0.5
**Naproxen**	2	0.5
**Rosuvastatin**	2	0.5
**Sulfasalazine**	2	0.5
**Ceftazidime**	1	0.2
**Clozapine**	1	0.2
**Duloxetine**	1	0.2
**Ganciclovir**	1	0.2
**Interferon**	1	0.2
**Itolizumab**	1	0.2
**Ketoconazole**	1	0.2

**Lamotrigine**	1	0.2
**Leflunomide**	1	0.2
**Methimazole**	1	0.2
**Olanzapine**	1	0.2
**Progestogens**	1	0.2
**Estrogens**	1	0.2
**Venlafaxine**	1	0.2
