# Supplementary material for: Does diabetes mellitus comorbidity increase the risk of drug-induced liver injury during tuberculosis treatment?
Source: PLoS One. 2023 May 31;18(5):e0286306. doi: 10.1371/journal.pone.0286306 (PMC10231779; doi:10.1371/journal.pone.0286306)
Supplement: S2 Table — 1 Cases: Alcoholic liver disease but not cirrhotic (1), steatosis (1), lithiasis (1), granulomatous hepatitis (1), Systemic Lupus Erythematosus (1) and choledocholithiasis (1). One patient had cirrhosis and congestive heart failure concomitantly. 1 Controls: Alcoholic liver disease but not cirrhotic (2), steatosis (1), autoimmune hepatitis (1), liver metastasis (1), non-alcoholic steato-hepatitis (1). (PDF) [file pone.0286306.s003.pdf]

S2 Table. Other liver diseases in cases and controls

Liver disease	Cases (n= 22)		Controls (n=16)	
	Frequency	%	Frequency	%
**Cirrhosis**	11	50	0	0
**Liver TB**	3	13.6	6	37.5
**Congestive heart failure**	1	4.5	3	18.8
**Others** [1]	6	27.3	6	37.5
**Unknown**	2	9.1	1	6.3

[1] Cases: Alcoholic liver disease but not cirrhotic (1), steatosis (1), lithiasis (1), granulomatous hepatitis (1), Systemic Lupus Erythematosus (1) and choledocolithiasis (1). One patient had cirrhosis and congestive heart failure concomitantly.

[1] Controls: Alcoholic liver disease but not cirrhotic (2), steatosis (1), autoimmune hepatitis (1), liver metastasis (1), non-alcoholic steato-hepatitis (1)
