# Supplementary material for: Does diabetes mellitus comorbidity increase the risk of drug-induced liver injury during tuberculosis treatment?
Source: PLoS One. 2023 May 31;18(5):e0286306. doi: 10.1371/journal.pone.0286306 (PMC10231779; doi:10.1371/journal.pone.0286306)
Supplement: S3 Table — CNS: central nervous system. 1 Cases: peritoneum (4), bone marrow (3), testicle (2), pericardium (1), bone (1), kidney (1) and psoas (1). 1 Controls: eye (7), pericardium (6), peritoneum (5), testicle (3), bone (2), skin (2), joint (1), kidney (1), bladder (1), ureter (1), oesophagus (1), larynx (1), pharynx (1), vertebrae (1), intervertebral disc (1), thoracic wall (1) and bone marrow (1). (PDF) [file pone.0286306.s004.pdf]

S3 Table. Extrapulmonary TB sites (not mutually exclusive) in cases and controls

Extrapulmonary site	Cases (n=72)		Controls (n=214)	
	Frequency	%	Frequency	%
**Pleura**	30	41.7	112	52.3
**Lymph nodes**	30	41.7	65	30.4
**CNS**	9	12.5	17	7.9
**Liver**	6	8.3	8	3.7
**Intestine**	5	6.9	3	1.4
**Spleen**	2	2.8	8	3.7
**Others [1]**	13	18.1	36	16.8
**Unknown**	0	0	1	0.5

CNS: central nervous system

[1] Cases: peritoneum (4), bone marrow (3), testicle (2), pericardium (1), bone (1), kidney (1) and psoas (1)

[1] Controls: eye (7), pericardium (6), peritoneum (5), testicle (3), bone (2), skin (2), joint (1), kidney (1), bladder (1), ureter (1), oesophagus (1), larynx (1), pharynx (1), vertebrae (1), intervertebral disc (1), thoracic wall (1) and bone marrow (1)
